# Effect of Surface Treatments with Low-Pressure Plasma on the Adhesion of Zirconia

**DOI:** 10.3390/ma16176055

**Published:** 2023-09-03

**Authors:** Pablo Sevilla, Mustafa Gseibat, Jesús Peláez, María J. Suárez, Carlos López-Suárez

**Affiliations:** Department of Conservative Dentistry and Buco-facial Prosthesis, Faculty of Odontology, University Complutense of Madrid, 28040 Madrid, Spain; pasevi01@ucm.es (P.S.); mam@ucm.es (M.G.); carlop04@ucm.es (C.L.-S.)

**Keywords:** zirconia, adhesion, alumina sandblasting, plasma treatment, shear bond strength

## Abstract

The purpose of this study was to investigate the effect of low-pressure plasma on the contact angle, shear bond strength (SBS), and the failure mode of zirconia ceramic. Zirconia specimens were divided into three groups according to the surface treatment methods as follows: sandblasting with aluminum oxide (ZR-C), sandblasting with aluminum oxide and oxygen plasma (ZR-CP), and argon plasma (ZR-P). The contact angle, SBS, and surface characteristics were tested after thermocycling. Data analysis was made using the Kruskal–Wallis test and one-way analysis of variance. Plasma treatment significantly reduced the contact angle (*p* < 0.001) with the lowest value for the Zr-P group. An increase in oxygen and a decrease in carbon was observed on the zirconia surface in both plasma groups. For the SBS, there were significant differences among the groups (*p* < 0.018), the Zr-CP group showing the highest bond strength. Mixed failures were the most frequent. Plasma treatment was effective in increasing the wettability, increasing the oxygen/carbon ratio without changing zirconia surface morphology. The sandblasting plus plasma with oxygen group exhibited the highest bond strength.

## 1. Introduction

High-strength ceramics have been developed as an alternative to metal–ceramic prostheses to improve the aesthetics and integration of restorations, both at dental and gingival level [1]. In particular, zirconium oxide (zirconia) provides high biocompatibility, low bacterial adherence, and good optical properties, thus achieving superior aesthetics and gingival health [2,3,4].

Zirconia is a polymorphic material, whose crystalline phase presents three allotropic forms depending on temperature: monoclinic, tetragonal, and cubic. Zirconia presents a monoclinic phase at room temperature. During the cooling of processed zirconia, the spontaneous transformation from the tetragonal phase to the monoclinic phase occurs, and is characterized by an increase in volume of approximately 4% that can lead to high tension and possible development of undesirable cracks [2,5,6]. Furthermore, when zirconia is subjected to a mechanical stress a phase transformation, from tetragonal to monoclinic, can also occur. This transformation process is called transformation toughening [6,7,8] and acts as a crack limiter [2,5].

To prevent phase transformation, zirconia must be stabilized by the addition of stabilizing oxides, such as yttrium, cerium, or magnesium [2,3,5,6,8]. Zirconia used in dentistry contains mainly tetragonal crystals partially stabilized with yttrium oxide [1,5,9]. Yttria-stabilized tetragonal zirconia polycrystalline (Y-TZP) has a flexural strength of 900 to 1400 MPa and a fracture toughness of 9 to 10 MPa/m^0.5^, making it the dental ceramic with the best mechanical properties [1,2]. Several variants of Y-TZP have been developed, depending on additives and stabilizers, and successive generations of zirconia have been introduced [3,8,9]. To date, four generations of zirconia have been developed based on its chemical structure, mainly due to its content of yttria and alumina [7]. The content of alumina (0.25–0.05%) influences zirconia translucency in such a way that the more the alumina content, the more opaque it will be, this being an ideal material for frameworks or full-contour restorations, mainly in the posterior sector [10].

Cementation is an important step in the clinical long-term success of the restorations [11]. However, despite the high popularity of zirconia restorations, an optimal cementation protocol has not been yet well stablished, making difficulties for clinicians regarding the correct technique or cement choice [1,12,13].

Zirconia full-coverage restorations with adequate retention and thickness can be cemented with convectional agents, such as zinc phosphate, glass ionomer, or resin-modified glass-ionomer cements. Resin-modified glass ionomer cement and self-adhesive resin cement demonstrated similar clinical outcomes in zirconia crowns [14]. However, most previous studies recommended adhesive cementation for zirconia restorations to ensure better retention and marginal fit. Furthermore, the zirconia surface needs to be prepared before applying the resin cement, since the pretreatments increase the bond strength. [1,12,13,15].

The most common zirconia bonding protocols consist of two parts: a surface pre-treatment and the application of a chemical bonding agent [13,15,16,17,18,19]. The objective of the pre-treatments is to increase the surface energy and wettability by generating roughness, sandblasting with aluminum oxide being the most extended method used [20]. Incorrect pre-treatment by means of increased aluminum oxide particle size, inadequate pressure, or application time can lead to crack formation and phase change, compromising the success of the zirconia restorations [21,22,23,24]. The introduction of plasma for surface treatment showed an enhancement of adhesion by improving the surface energy of the material without increasing the roughness, and therefore causing less damage than alumina particles [21,25,26,27,28]. However, several factors influence the results, such as the type of gas or the exposure time [28,29].

To date the resin-bonding protocol for zirconia restorations is still controversial. Therefore, the aim of the present study was to evaluate and compare the influence of low-pressure plasma on the contact angle, the shear bond strength of zirconia ceramic, and the failure mode. The null hypothesis tested was that surface treatment by plasma would not affect the adhesion of zirconia.

## 2. Materials and Methods

### 2.1. Preparation of Specimens

Eighty-four cylinders (12 mm diameter × 9 mm height) were fabricated from a zirconium oxide disc (IPS e.max ZirCAD LT; Ivoclar Vivadent, Schaan, Liechtenstein) on a milling machine (Zenotec Select, Wieland Dental, Pforzheim, Germany), and subsequently sintered in a furnace (Astromat µSic Dekema, Dental-Keramikofen GmbH, Freilassing, Germany) according to the manufacturer’s instructions. The samples were ultrasonically rinsed with distilled water for 5 min and stored in plastic boxes, that were cleaned with 99.9% isopropyl alcohol (EQM, Madrid, Spain) and subjected to a cleaning cycle with 100% oxygen plasma for 1 min [28].

### 2.2. Specimen Treatments

**Figure 1:**
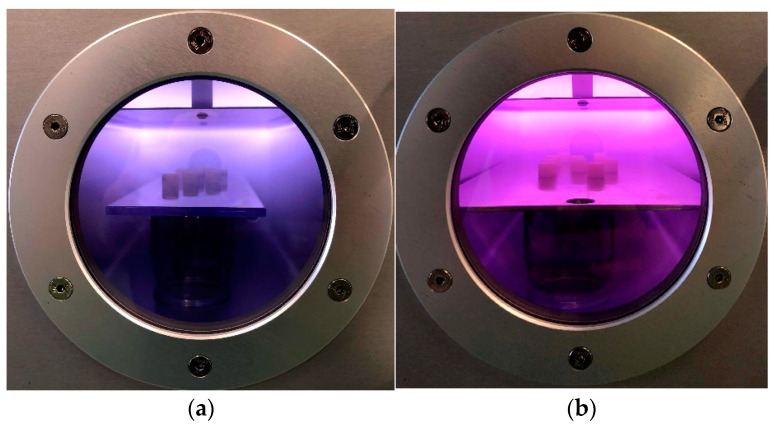
Surface treatment with plasma: (**a**) plasma oxygen; (**b**) plasma argon.

**Figure 2:**
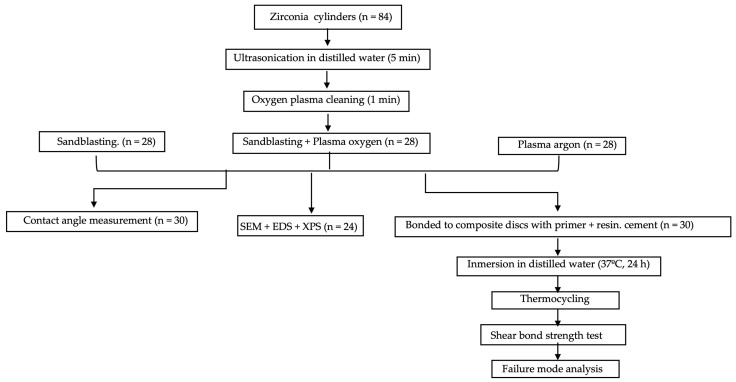
Flowchart of the specimen preparation and experimental process.

**Figure 3:**
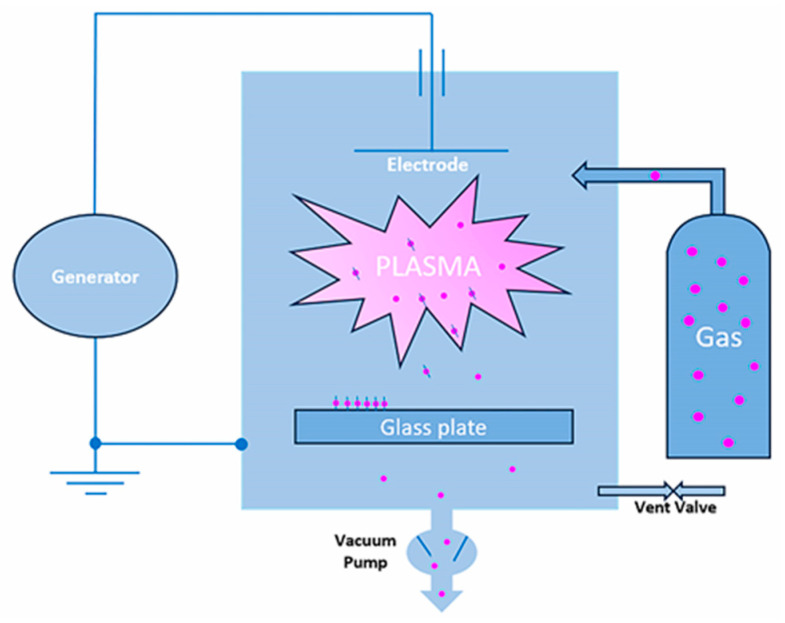
Schematic diagram of the plasma system employed.

The specimens were numbered and randomly divided into 3 groups (*n* = 28 each), using a randomization list (www.alazar.info, 7 October 2019). The sample size was determined using a software program (G*Power 3.1.9.4, Samsøvej, Denmark) revealing a total sample size of 30 with an effect size of 0.8 at 0.8 power and a significance level of 0.05. The groups were categorized according to surface treatment: sandblasting with aluminum oxide (Zr-C); sandblasting with aluminum oxide plus plasma with oxygen (Zr-CP); and plasma argon (Zr-P) (Figure 1). Figure 2 summarizes the overall process, and the details of the treatments, and the instruments used are described in Table 1 and Table 2 and Figure 3.

### 2.3. Contact Angle Measurement

Thirty cylinders were used to measure the contact angle in each group (*n* = 10) with a drop shade instrument (FTA 1000 B Class, First Ten Angstroms Inc., Portsmouth, VA, USA) connected to a video camera, by the sessile drop technique [27,28,29,30,31], in order to evaluate the wettability, after dropping a small amount of water (3 µL) at 3 different locations of the disc on each specimen. The images obtained were analyzed using image analysis software (FTA 32 2.0, First Ten Angstroms Inc.), and the average contact angle was obtained. The measurements were performed at a constant temperature (22 °C) and relative humidity (30 ± 10%) [28].

### 2.4. Bonding Procedure

Thirty hybrid composite (Tetric Evoceram Bulk Fill, Ivoclar Vivadent) discs (4 mm in diameter × 5 mm in length) were fabricated from a transparent silicone mold (Exaclear, GC, Tokyo, Japan), which were light polymerized (Bluephase, Ivoclar Vivadent). Composite discs were cemented to zirconia cylinders following the manufacturer’s procedure. A universal primer (Monobond Plus, Ivoclar Vivadent) was applied to the ceramic surface with a micro-brush for 1 min. Subsequently, the composite discs were cemented using a self-adhesive resin cement (Multilink Automix, Ivoclar Vivadent). To standardize the cementation process, a special aluminum device was fabricated. After positioning the samples, the cement was applied and the composite discs were then bonded to the zirconia surface. Excess cement was removed by a micro-brush, and light cured with a LED light-curing unit (Bluephase, Ivoclar Vivadent) by focusing the light perpendicular to the adhesive interface in 4 directions (30 s on each side at 600 mW/cm^2^). The samples were stored in distilled water at 37 °C for 24 h and then subjected to thermocycling.

### 2.5. Shear Bond Strength (SBS) Test and Failure Analysis

The cemented specimens (*n* = 10 per group) were placed into a polyethylene container with artificial saliva Fusayama-Meyer (LCTech, Obertaufkirchen, Germany), and aged by thermocycling for 6000 cycles at 5 °C and at 55 °C with a 30 s dwell time in a climatic chamber (CCK0/81; Dycometal, Barcelona, Spain) [32].

The SBS measurement method followed the International Organization for Standardization (ISO 290022:2013) [33]. The specimens were subjected to a shear load at 1 mm/min crosshead speed using a testing machine (TA.XTPlus, Stable Micro Systems, Godalming, UK) until debonding. The bond strength values were recorded in MPa. After the SBS test, the fractured superficies were evaluated and classified according to the failure type: adhesive, cohesive, and mixed [25,27,29], using a stereomicroscope (Leica MZ12, Leica Microsystems, Wetzlar, Germany) at 10× magnification.

### 2.6. Scanning Electron Microscope (SEM), Energy Disperse Spectroscopy (EDS), and X-ray Photoelectron Spectroscopy (XPS)

The surface morphology of eight zirconia samples from each group was examined using a SEM (6400 JSM, JEOL, Tokyo, Japan) at 5000× magnification, after surface treatments. Additionally, EDS was used to identify and quantify elemental composition of the treated areas of specimens [29]. XPS was also performed by spectroscopy (VG Microtech MT500, Fisons Instruments, Glasgow, UK) to determine the carbon and oxygen atomic percentages and the peak of each element.

### 2.7. Data Processing

Statistical analysis was performed with statistical software (SPSS 25.0, SPSS Inc., Chicago, IL, USA). Descriptive statistics, including the mean and the standard deviation (SD), were calculated for each group. The normality of the variables was evaluated by the Shapiro-Wilk test. For the contact angle variable, given the non-normality of data, the Kruskal–Wallis test was applied. The SBS was analyzed by one-way analysis of variance (ANOVA) and Tukey’s HSD post hoc test. The level of significance was set at α = 0.05.

## 3. Results

### 3.1. Contact Angle Measurement

Significant differences were found in the contact angle (*p* = 0.001) The lowest contact angle values were found in the Zr-P group (15 ± 7)°, with statistically significant differences with the Zr-C group (55 ± 19)° (*p* = 0.013) and the Zr-CP group (33 ± 6)° (*p* = 0.001). No differences were observed between the Zr-C and the Zr-CP group.

### 3.2. Shear Bond Strength and Failure Mode Analysis

The SBS values showed significant differences among the groups (F = 4.674; *p* = 0.018). The highest SBS values were observed in the Zr-CP group and were significantly higher than the Zr-C group. However, no differences were observed between the Zr-C and the Z-P groups (Table 3).

**Figure 4:**
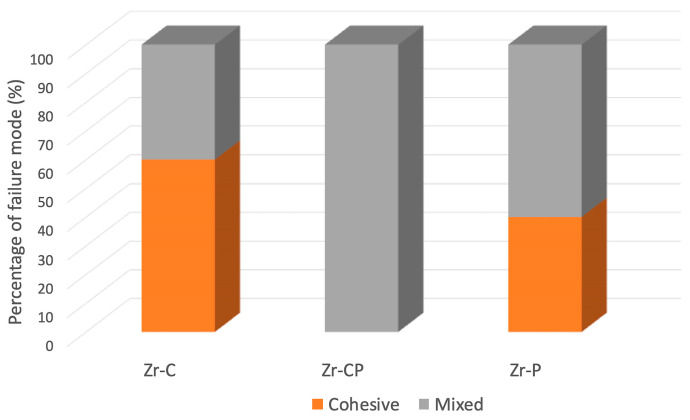
Percentage of failure mode for each group. Zr-C, sandblasting; Zr-CP, sandblasting + plasma oxygen; Zr-P, plasma argon.

Two types of failure were observed: cohesive ad mixed. Mixed failures were the most prevalent in both groups treated with plasma (100% in Zr-CP group and 60% in Zr-P group), while the Zr-C group mainly exhibited cohesive failure (60%). Adhesive failures were not observed in any of the groups (Figure 4).

### 3.3. SEM, EDS, and XPS

**Figure 5:**
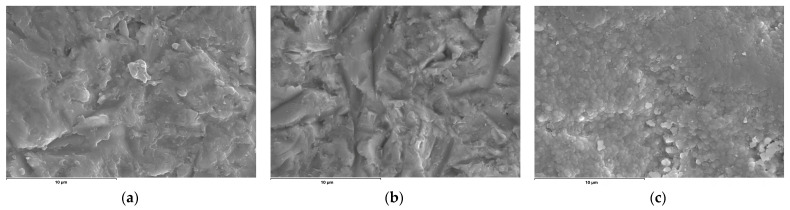
Representative SEM images (5000×) of the zirconia for each surface treatment: (**a**) Zr-C, sandblasting; (**b**) Zr-CP, sandblasting + plasma oxygen; (**c**) Zr-P, plasma argon.

Regarding the morphological study, it was observed that the groups treated with sandblasting (Zr-C and Zr-CP) had an irregular topography with a slight roughness formed from short, shallow grooves, while the group Zr-P showed a smooth, regular surface without any type of roughness (Figure 5).

**Figure 6:**
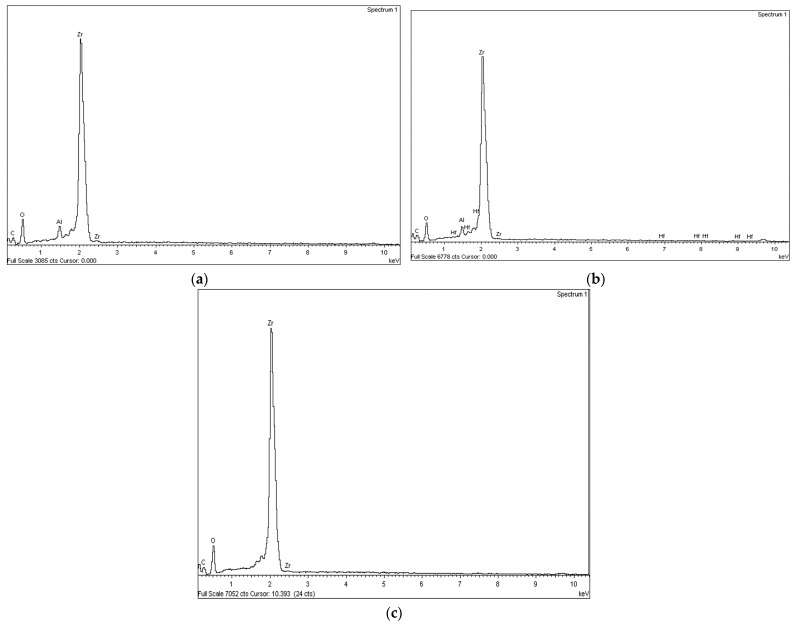
Energy Disperse Spectroscopy of zirconia surface after each surface treatment. (**a**) Zr-C, sandblasting; (**b**) Zr-CP, sandblasting + plasma oxygen; (**c**) Zr-P, plasma argon.

The chemical analysis of the treated surfaces showed that there were hardly any changes in the elemental composition of the zirconia ceramic. Small increases of aluminum were recorded in the sandblasted group. No argon was detected in the Zr-P group. An increase of oxygen was observed in all groups after surface treatment, mainly in the Zr-P group (Figure 6).

XPS analysis revealed different carbon oxygen (O) and carbon (C) ratios in the groups, increasing the O/C ratio in both plasma groups. Likewise, an increase in oxygen content and a decrease in carbon content was observed in the Zr-CP (0.26) and Zr-P (0.15) groups (Table 4).

## 4. Discussion

The present study evaluated the effect of surface treatment of zirconia by low-pressure plasma on the contact angle and the SBS. The data obtained support the rejection of the null hypothesis, as the contact angle and SBS exhibited significant differences.

Although zirconia is widely used in prosthodontics, an optimal resin-bonding protocol for zirconia restorations is still a challenge, and it should be noted that clinical recommendations are mainly based on in vitro studies [1,13,29]. Sandblasting with alumina is the most extensive zirconia surface treatment [13,15,19,31,34,35,36]. However, possible damage on the zirconia surface has been reported [21,27,29,37,38], and alternative methods have been proposed, such as plasma, to enhance the adhesion to zirconia [13,25,39].

In the study, the contact angle decreased in both plasma groups but with significant differences only for the Zr-P group, which decreased by 73% compared to the Zr-C group, indicating an increase in the hydrophilicity of the zirconia surface. These results are consistent to those obtained by other studies [25,27,29,30,40,41,42]. The contact angle is a frequent way to calculate the surface energy (SE) indicating the wettability of a surface [30,42]. Valverde et al. [25] suggested that the results were related to an increase of oxygen atoms in the surface which leads to higher polarity. The increased hydrophilicity of zirconia surface after plasma treatment improves the SE and wettability [27,29,30]. Furthermore, it has been reported that a strong correlation exists between contact angle and the adhesion strength to zirconia [43].

SEM evaluation revealed no significative morphological changes after plasma argon treatment, which showed a smooth surface. However, the Zr-C and the Zr-CP showed an irregular topography and slight roughness. The results agree with previous studies [29,30,44]. The chemical evaluation by EDS showed no significant morphological changes in the composition of zirconia after surface treatment except for a small increase of aluminum in the Zr-C group and an increase of oxygen in all the groups, mainly in the Zr-P group. The results clearly indicate that plasma treatment did not affect the surface topography or morphological properties of zirconia restorations, consistent with previous studies [29,45].

The reason for contact angle reduction in the plasma treated groups can be explained by the XPS analysis. The results revealed and increase in O and a decrease in C in both plasma groups compared to the Zr-C group. Moreover, the O/C ratio increased in both plasma groups, mainly in the Zr-P group. The increase in the atomic percentage of O improves zirconia surface hydrophilicity. The results were consistent with previous studies [25,38,44,45,46,47,48]. The lower values of contact angle correspond with higher values of O and lower values of C, indicating an enhancement in the wettability, as previously reported [38]. No Ar was observed in the Zr-P group, indicating that no Ar was integrated into the zirconia surface.

The plasma treatments evaluated in the study increased the shear bond strength in both plasma groups compared to the control group, with the highest adhesion strength obtained by the Zr-CP followed by the Zr-P group, with no differences between both plasma groups and between Zr-C ad Zr-P groups. In addition, the groups evaluated obtained values higher than 10 MPa, which is considered the clinical acceptability threshold [49]. However, the results indicated that the argon group obtained similar SBS values to the sandblasted group, and the combination of sandblasting plus plasma was the most effective surface treatment for the bonding strength. Several in vitro studies investigated the bond strength between zirconia and resin cements, results indicating that plasma treatments enhance the bonding strength [25,29,30,38,39,42,50]. Ito et al. [41] and Tabari et al. [30] reported that non-thermal atmospheric pressure plasma improves the bonding strength as effectively as alumina sandblasting, the results of the present study agreeing with Egoshi et al. [42] with non-thermal low-pressure plasma and self-adhesive cement, and Valverde et al. [25] with atmospheric argon plasma, obtaining the highest bond strength in the abrasion plus plasma specimens. Conversely, Kim et al. [27] reported lower SBS values in the plasma group compared to sandblasted or sandblasted plus plasma after thermocycling. In the same way, Ahn et al. [31] obtained a significantly higher SBS value in the sandblasted group plus primer than the plasma groups, although sandblasting plus plasma plus primer showed a positive effect on the SBS. Furthermore, Lümkermann et al. [48] concluded that plasma treatment cannot substitute for sandblasting to the zirconia bonding. Moreover, a previous study reported similar SBS values to those obtained in the present study for the Zr-CP group, with the same primer and cement for specimens subjected to tribo-chemical silica coating with Silk-Jet powder [51].

Differences among the studies may be due to several factors. Different tests have been used to evaluate the bond strength between zirconia and resin cement, probably due to the lack of an international standard. Macroshear was the most widely used test, as in the present study, probably due to its simplicity [13]. However, other authors employed the micro-shear test, and this variability among tests make it very difficult to compare results among the studies. In addition, studies on plasma as zirconia surface treatment showed great heterogeneity in the methodology employed. The main difference among the studies was the means of generating the plasma and its application, in terms of the type of device, the exposure parameters (time, energy, pressure), or the gas employed. Several gases have been investigated, with argon and oxygen as more frequent. However, to date it is not clear which gas obtains the best results [28,30,44]. The plasma device used in the study is easy to use and was the same for both plasma gases used, due to its two independent channels. The expense generated and maintenance is low. The time used was that recommended by the company and was also based in a previous study [28]. Although the treatment time was different in Zr-CP and Zr-P groups, the samples’ temperature was similar after the treatment and it can be handled without any risk.

On the other hand, the type of cement also affects the SBS, as previously reported [27,31,42,48]. However, adhesive protocol differs widely among the studies. Most authors use self-adhesive resin cements with or without the prior application of a ceramic primer [25,27,29,30,31,35,39,42,48,52,53]. Furthermore, previous studies indicated that the use of functional monomers, such as 10-methacryloyloxydecyl dihydrogen phosphate (MDP), is advantageous for a strong bond to zirconia [12,13,27,48,53,54]. However, one important problem is that there is great variability in the percentage of the components and the viscosity of the cements, and that the manufacturers do not give information on the exact composition [12,13,27,31]. Kim et al. [27] suggested in their study that the difference in the SBS may be due to the monomer characteristics of the cements employed. In the present study, a zirconia primer containing MDP and a self-adhesive cement without acidic monomers was used, based on the previous recommendations that could influence the results.

With regards to the failure mode, mixed failures were the most prevalent in both plasma groups, and a unique type of failure in the Zr-CP group. The results were consistent with previous findings [9,27,31,42,44]. The cohesive failure was the second most prevalent in both zirconia groups, consistent with Liu et al. [29], who reported that, after plasma treatment time increased, adhesive fracture significantly decreased. Conversely, other authors did not report cohesive fractures [27,42,44]. In the study, adhesive failures were not observed, which could indicate that plasma treatment contributed to adhesive bonding, as previously reported [42]. Results of the failure mode were consistent with the results of the SBS test, given that the Zr-CP obtained the highest SBS values and 100% of mixed failure. Comparing studies, however, is difficult due to methodological differences, and there is a need for a standardized methodology.

The results of the study indicated that the plasma treatment used increased the hydrophilicity and wettability of the zirconia surface, mainly with the argon gas. However, these results seem not to be the principal cause of increased bonding strength, and a previous study suggested that SBS and contact angle does not show an exact correlation [44]. Furthermore, the findings of the study suggest that the zirconia smooth surface after plasma surface treatment could play an important role in the zirconia adhesion to chemical agents compared to sandblasted zirconia surface, as previously reported [55]. The results of this study demonstrated that the plasma treatments evaluated and the plasma device used can be a recommendable option for improving zirconia adhesion, without modifying the zirconia surface.

The study was limited by the use of only one type of cement and because no surface roughness measurement was done. Further studies must be carried out evaluating different cements and different plasma sources and treatment times. In addition, the study was performed in vitro under controlled conditions that could not reflect the clinical situation. Therefore, long-term clinical studies are necessary to confirm the effectiveness of plasma treatment on the adhesion to zirconia.

## 5. Conclusions

Within the limitations of this study, non-thermal low-pressure plasma improved the adhesive properties of zirconia, without deteriorating or modifying the surface. Both gases evaluated increased the zirconia surface wettability. Plasma treatment increased the amount of oxygen and decreased the amount of carbon on the zirconia surface. The combination of sandblasting with aluminum oxide plus oxygen plasma obtained the highest bond strength. Mixed failures were the most frequent in plasma groups.

## Figures and Tables

**Table 1:** Surface treatment groups.

Group	Surface Treatment
Zirconia Control (Zr-C)	Sandblasting 50 µm Alumina at 2 bar, at 10 mm for 15 s
Zirconia Control + Plasma (Zr-CP)	Sandblasting 50 µm Alumina at 2 bar, at 10 mm for 15 s + Plasma Oxygen 100%, 0.25–0.30 mbar, 40 kHz, and 1000 W for 5 min
Zirconia Plasma (ZR-P)	Plasma Argon 100%, 0.25–0.30 mbar, 40 kHz, and 1000 W for 60 min

**Table 2:** Instruments used for surface treatments.

Instrument	Type	Features	Manufacturer
PICO PCCE	Low-pressure plasma system	Regular chamber and PC control. Low-frequency plasma generator: 40 kHz, 1000 W Rectangular electrode inside the chamber	Diener Electronic GmbH, Ebhausen, Germany.
Constellation Practic	Sandblaster	Working pressure 2–6 bar, Voltage 230 V, 50/60 Hz, Power consumption 0.4 W, Nozzle 0, 75 mm	Mestra, Bizkaia, Spain.

**Table 3:** Means, standard deviations (SD), minimum and maximum shear bond strength values (MPa) for each group.

Group	*n*	Mean ± SD	Min	Max
Zr-C	10	13.4 ± 1.9 ^a^	6.5	21.9
Zr-CP	10	21.3 ± 1.8 ^b,c^	11	29
Zr-P	10	15.3 ± 2 ^a,c^	6.4	28

Note: Groups with the same lowercase letter were not significantly different.

**Table 4:** Atomic percentage of each element and O/C ratio for each group, calculated by X-ray photoelectron spectroscopy (XPS).

Group	O 1s	C 1s	Zr 3d	Y 3d	F 1s	Na 1s	Al 2p	Si 2p	O/C
Zr-C	42.9	32.2	7.7	0.6	2.3	0.9	13.3	-	1.3
Zr-CP	56.8	15.2	9.7	0.6	1.7	1.2	14.7	-	3.7
Zr-P	64.9	9.8	15.2	2.1	-	-	-	7.8	6.7

## Data Availability

No new data were created or analyzed in this study. Data sharing is not applicable to this article.
